# Innate airway responses shape permissiveness to human respiratory syncytial virus

**DOI:** 10.1016/j.virusres.2026.199734

**Published:** 2026-04-27

**Authors:** Laura L.A. van Dijk, Laurine C. Rijsbergen, Alexander C. Havelaar, Yvette den Hartog, Rosanne W. Koutstaal, Kevin Groen, Rory D. de Vries, Rik L. de Swart

**Affiliations:** Erasmus MC, University Medical Centre Rotterdam, Netherlands

**Keywords:** Host response, Microbiome, Viral interference, Co-infection, Tissue damage

## Abstract

The interaction between human respiratory syncytial virus (HRSV) and the innate immune system has been demonstrated both *in vitro* and *in vivo*. Disruption of interferon (IFN) signaling pathways increases susceptibility and permissiveness to HRSV infection, whereas pretreatment of cells with IFN confers (partial) resistance. This suggests that HRSV disease severity is likely influenced by a pre-existing antiviral state of the respiratory epithelium, driven by baseline or primed expression of type I and type III IFNs.

Here, we investigated whether prior exposure to respiratory bacteria or viruses alters *in vitro* susceptibility and permissiveness to HRSV infection by shaping an antiviral state using both immortalized cell lines and airway organoid models. In A549 cells, pre-exposure to *S. aureus* had the most significant impact by reducing HRSV infection and inducing robust interferon responses. However, this effect was not reproduced in airway organoids. Conversely, sequential virus infection experiments in airway organoids revealed that prior infection with human parainfluenza virus type 3 (HPIV‑3) reduced the spread of subsequent HRSV infection. In addition to interferon signaling this proved to be associated with epithelial damage mediated by HPIV-3 infection.

Collectively, these findings show that HRSV susceptibility and permissiveness are influenced by the up- or downregulation of specific anti- and pro-viral factors induced by prior bacterial or viral exposure, together with the maintenance or disruption of epithelial integrity. Understanding these interactions could be crucial when identifying specific risk groups for severe HRSV-associated disease and the development of targeted HRSV interventions.

## Highlights

• Pre-existing antiviral states can shape permissiveness to HRSV.
• Prior microbial exposure can either decrease or increase HRSV infection.
• Prior HPIV-3 infection reshapes epithelial defenses and architecture during subsequent HRSV infection.

## 1 Introduction

Infections with human respiratory syncytial virus (HRSV) are a major cause of bronchiolitis and pneumonia in infants, immunocompromised patients and frail elderly. HRSV is estimated to be responsible for at least 33 million acute lower respiratory tract infections yearly, contributing to over 100,000 fatal cases (Li et al., 2022). Prophylactic options include monoclonal antibodies (palivizumab and nirsevimab) for infants and vaccines for older adults and pregnant women (Alandijany and Qashqari, 2025; Nayak and Quinn, 2025).

When HRSV enters a susceptible host, innate pattern recognition receptors (*e.g.* RIG-I and toll-like receptors (TLRs)) are triggered, leading to the production of type I and III interferons (IFN) by epithelial cells or other innate immune cells (Johansson, 2016). The type I IFN-α and IFN-β bind to the IFN-a/b receptor (IFNAR, composed of two subunits IFNAR1 and IFNAR2), present on virtually all nucleated cells, in a paracrine or autocrine fashion. Type I IFNs are produced rapidly after viral infection, leading to the generation of a pro-inflammatory environment with antiviral potency (Voigt and Yin, 2015). The type III IFN-λ binds to the IFN lambda receptor (IFNLR, consisting of two subunits IFNLR1 and IL-10R2) and shares many properties with type I IFN. However, IFNLR expression is restricted to mucosal epithelial cells (Li et al., 2009; Voigt and Yin, 2015), suggesting a specialized role of type III IFNs in mucosal immunity.

IFN receptor engagement activates two major intracellular signaling pathways, referred to as the canonical and non-canonical pathways, with signal transducer and activator of transcription 1 (STAT1) modulating both pathways (Rauch et al., 2013). This triggering leads to abundant expression of IFN-stimulated genes (ISGs) that limit virus replication (Schneider et al., 2014). Collectively, these responses are referred to as the induction of an antiviral state. This antiviral state plays a critical role in restricting respiratory virus infections. In mice, disruption of type I and III IFN signaling pathways increases the impact of HRSV infection, demonstrated by higher lung viral load and weight loss (Goritzka et al., 2014). Inherited deficiencies in innate immune pathways, such as genetic variants affecting TLRs or IFN‑signaling genes, are associated with significantly increased severity of HRSV disease due to impaired early antiviral responses (Janssen et al., 2007). Likewise, individuals with neutralizing autoantibodies against type I interferons may also suffer severe HRSV-associated disease, as these autoantibodies compromise critical IFN-mediated antiviral functions by neutralizing key cytokines that control replication (Hijano et al., 2019; Hale, 2023; Groen and Hale, 2026). Among infants infected with HRSV, those with mild symptoms exhibited higher nasopharyngeal concentrations of type I and type III IFN during infection compared with those who developed more severe disease (Taveras et al., 2022).

Intrinsic or environmentally primed antiviral states are considered key determinants of HRSV disease outcome. Different factors shape such antiviral states, including irritants, allergens, or genetic predisposition (Galli et al., 2008; Moldoveanu et al., 2009; Kenney et al., 2017). Another important determinant of disease outcome is the composition of the resident respiratory microbiome: several epidemiological studies have reported associations between microbiome composition and HRSV disease severity (Duttweiler et al., 2004; Thorburn and Van Saene, 2007; Yoshida et al., 2013; Nguyen et al., 2015; de Steenhuijsen Piters et al., 2016; Kristensen et al., 2024). In particular, presence of *Streptococcus pneumoniae* or *Haemophilus influenzae* has been associated with more severe HRSV-associated disease (O'Brien et al., 2009; Watt et al., 2009; Zhang et al., 2020), but also *Pseudomonas aeruginosa* was associated with an increased risk of developing HRSV bronchiolitis (Thorburn et al., 2006). On the other hand, the presence of *Corynebacterium species* and *Lactobacillus rhamnosus* was linked to a reduced risk of severe HRSV disease (Tomosada et al., 2013; Kanmani et al., 2017; Man et al., 2019). Finally, *Staphylococcus aureus* was associated with fewer disease-related hospitalizations (de Steenhuijsen Piters et al., 2016).

These observations suggest that the airway microbiome actively influences host immune responses during HRSV infection. One potential mechanism by which bacteria can modulate HRSV pathogenesis is through the regulation of cytokine expression in the airway microenvironment. Recognition of bacterial components by TLRs expressed by respiratory epithelial and immune cells triggers signaling cascades that lead to pro-inflammatory cytokine production (Mogensen, 2009; Moresco et al., 2011). Several studies assessed how the cytokine responses induced by microbiome taxa influence HRSV infection *in vitro* and *in vivo*. Pretreatment of a bronchial epithelial cell line (BEAS-2B) with heat-inactivated *H. influenzae*, a bacterium linked to more severe disease in infants, followed by HRSV infection resulted in a synergistic increase in IL-6 and IL-8 production, compared to cells infected with HRSV alone (Gulraiz et al., 2015; Bellinghausen et al., 2016). Moreover, HRSV replicated more efficiently in cells pre-exposed to *H. influenzae* than unexposed cells (Gulraiz et al., 2015). Inoculation of mice with viable *C. pseudodiphtheriticum,* a *corynebacterium spp.,* induced the production of TNF-α, IL-6, IFN-λ, IFN-β and IL-10, measured in both serum and bronchoalveolar lavage (BAL), which was then associated with reduced lung HRSV titers (Kanmani et al., 2017). Combined, this shows that different bacteria can prime the respiratory microenvironment in different ways, rendering respiratory epithelial cells either more or less susceptible (reducing viral entry) or permissive (reducing viral replication) to HRSV infection.

In addition to the resident microbiome, prior viral exposures or co-infections could be another determinant. Even though less studied, the concept of viral interference describes how infection with one virus reduces infection with or replication of a subsequent virus (Du et al., 2022). Previous studies have shown that prior influenza A virus infection inhibited HRSV replication in mice and *in vitro* human airway epithelial cultures, associated with high levels of type I and type III IFN (Gilbert-Girard et al., 2025). Similarly, influenza A virus infection also suppressed severe acute respiratory syndrome coronavirus-2 (SARS-CoV-2) replication in nasal human airway epithelial cultures (Gilbert-Girard et al., 2024), and HRSV replication was negatively correlated with prior infection with human rhinovirus (HRV) by spatial-temporal modeling (Zhang et al., 2025).

This study aimed to elucidate how IFN responses induced by bacterial or viral stimuli influence HRSV infection and replication. First, we studied the role of type I and III IFN signaling using type I and III IFN receptor knockout cell lines and IFN pretreatment conditions. Subsequently, we examined the effects of prior exposure to distinct bacterial species or viral infections on HRSV susceptibility and replication in immortalized cell lines and airway organoids.

## 2 Materials and methods

### 2.1 Cells

A549 cells were cultured in Ham’s Nutrient Mixture F12 (21,765, Gibco Invitrogen, USA). HEp-2 cells were cultured in DMEM (12–733F, Lonza, BioWhittaker, Switzerland). Media were supplemented with 10 % heat-inactivated fetal bovine serum (FBS) (Sigma Aldrich, USA), 10 mM L-glutamine, 100 U/mL penicillin and 100 µg/mL streptomycin (together abbreviated as PSG) (Lonza BioWhittaker, Switzerland). Cells were cultured at 37 °C with 5 % CO_2_. Human adult bronchiolar organoids were cultured and differentiated as described previously (Rijsbergen et al., 2021). In short, non-tumor lung tissue from two lung-cancer surgery patients was used to obtain adult human lung stem cells from the distal airways. Every 10–14 days, airway organoids (AO) grown in 3D in matrigel were dissociated into single cells using tryplE express (Gibco) and split 1:6 to 1:12. AO were seeded and differentiated on transwell inserts coated with rat tail collagen type I (Corning), and subsequently grown in a 1:1 mixture of AO medium:complete base medium (CBM; Stemcell Pneumacult-ALI medium) until 100 % confluent. Next, medium was replaced with CBM only and cells were put at air-liquid interface (ALI). Medium was changed every 5 days; after 6 weeks cells were fully differentiated into pseudostratified ciliated epithelial cells and ready for use in experiments. Apical-out airway organoids (Ap-O AO) were generated as described previously (van Dijk et al., 2024). Briefly, 120,000 single cell organoids in 1 ml Pneumacult Ap-O AO medium (Stemcell technologies) were added to Aggrewell plates pre-coated with anti-adherence rinsing solution (Stemcell technologies). Over the next 2–4 days, cells were allowed to aggregate into small clumps. Subsequently, 3D structures were transferred to pre-coated flat bottom 24 well plates and further differentiated for 10–14 days with partial medium change every 2–3 days.

### 2.2 Production of knockout cells

Knockout (KO) A549 cells (ΔIFNAR1, ΔIFNLR1) were generated using Lentivector TLCV2 (Addene). Two sgRNAs per target gene were independently cloned into vector TLCV2 (IFNAR1 sgRNA1: 5′-GACCCTAGTGCTCGTCGCCG-3′, sgRNA2: 5′-ACAGGAGCGATGAGTCTGTC-3′, IFNLR1 sgRNA1: 5′-TCGCGCCACCGTCTACGGGT-3′, sgRNA2: 5′-ACTGAACGTGTAGATGGTTC-3′). Lentiviruses were produced by transfecting 293T cells at 70–80 % confluence in 10 cm tissue culture dishes with 2.8 µg plasmid VSV-G (Addgene), 9.2 µg of plasmid pBS-CMV-gagpol (Addgene), and 10 µg of plasmid TLCV2 with sgRNA sequences targeting IFNAR1 or IFNLR1 using FuGene HD transfection reagent (Promega, E2311) according to the manufacturer’s protocol. Cell supernatants were harvested 48 h post transfection and cell debris was removed by centrifugation for 3 min at 2000×*g*. For each knockout cell line, 2 mL of lentivirus-containing supernatant supplemented with 8 µg/mL polybrene was added to 1E5 A549 cells seeded in 6-well tissue culture plates were and incubated for 24 h. Medium was replaced for fresh DMEM supplemented with 10 % FBS, 1 % Pen/Strep, and doxycycline to induce Cas9 protein expression for 48 h. Transduced cells were selected using puromycin for 48 h and cells were subjected to limiting dilution to obtain single cell clones. Unmodified A549 cells were used as the wild-type (WT) control in all experiments comparing knockout cell lines.

### 2.3 Viruses

Generation and culture of the recombinant subgroup A clinical isolate-based HRSV strain, rHRSV^A11^EGFP(5), was described previously (Rennick et al., 2020). A clinical isolate of HPIV-3 was obtained as part of a healthcare worker study (approved by the Medical Ethics Committee of Erasmus MC, MEC-2024–0228). HPIV-3 stocks were grown on AO at ALI and harvested in Dulbecco’s phosphate-buffered saline supplemented with Ca and Mg (0.9 mM MgCl2 and 0.49 mM CaCl_2_, referred to as DPBS++) at 2–4 days post infection (DPI).

### 2.4 Detection of pSTAT1 by western blot

Recombinant human IFN-β (PeproTech) and recombinant human IFN-λ1 (PeproTech) were dissolved in distilled water (dH2O) containing 0.1 % (v/v) BSA (Sigma), aliquoted and stored at −20 °C until use. A549 cells (WT and KO) were seeded into a 24-well plate (Corning) at 150,000 cells per well and were treated with 5 ng/ml IFN-β or IFN-λ1 for 4 h. After treatment, cells were washed twice in ice-cold PBS, and 200 µl ice-cold RIPA buffer (Fisher Scientific) containing protease inhibitors (Fisher Scientific) and phosphatase inhibitors (Fisher Scientific) was added for 5 min on ice. Cells were detached by scraping and incubated for an additional 30 min at 4 °C while rotating. Cell lysates were centrifuged for 15 min at 20,000×g and stored at −80 °C until further analysis. For western blot, lysates were diluted to a final concentration of 1× NuPAGE loading dye (Bio-Rad) containing 5 % (v/v) 2-mercaptoethanol and SDS-PAGE analysis was done using precast AnykD TGX gels (Bio-Rad). Gels were run in tris-glycine SDS buffer (TGS) at 150 V for 30 min. Protein transfer was performed at 200 mA for 60 min onto 0.45 µm activated Immobilon-FL PVDF membranes (Millipore) in 1× TGS containing 20 % (v/v) methanol. Membranes were washed in 0.1 % (v/v) Tween in PBS (PBS-T) and blocked in PBS-*T* + 10 % (w/v) milk powder. STAT1 was detected with monoclonal mouse-anti-STAT1 (1:200, Santa Cruz) followed by HRP-conjugated polyclonal rabbit-anti-mouse IgG (1:1000, Dako). Phosphorylated STAT1 (pSTAT1) was detected with monoclonal mouse-anti-pSTAT1 (1:1000, Santa Cruz) followed by HRP-conjugated polyclonal swine-anti-rabbit IgG (1:1000, Dako). β-actin was detected using HRP-conjugated monoclonal mouse-anti-β-actin (1:5000, Santa Cruz). ECL Prime western blotting detection reagent (Amersham) was used as a substrate for chemiluminescence. Western blots were scanned on the GelDoc XR (Bio Rad) and analyzed using Adobe Illustrator 2022 version 26.31.

### 2.5 Quantification of ISG expression by RT-qPCR

A549 cells (WT and KO) were seeded into 24-well plates (150,000 cells per well) and incubated overnight at 37 °C with 5 % CO2. A549 cells were treated with 0.5 ml of 5 ng/ml IFN-β and/or IFN-λ1 (2.5 ng/well) in Ham’s-F12 growth medium. After overnight treatment, A549 cells were washed with phosphate-buffered saline (PBS). Subsequently, cells were lysed in 150 µl MagNA Pure 96 External Lysis Buffer (Roche) for 5–10 min. Cell lysates were added to a 96-well plate containing magnetic beads (AMPure XP beads; Beckmann Coulter), thoroughly mixed, and incubated for 20 min at room temperature. The 96-well plate was placed on a magnetic block (DynaMag™−96 side skirted magnet; Thermo Scientific) for 3 min to separate beads. Supernatants were carefully removed, and the beads were washed three times for 30 s with 200 µl 70 % (v/v) ethanol. After the third wash, ethanol was removed, and the beads were air-dried for three minutes at room temperature. The plate was removed from the block, and the beads were thoroughly resuspended in 50 µl dH2O. Plates were incubated for 20 min at room temperature to elute the RNA and subsequently transferred to the magnetic block. The dH2O (without beads) containing RNA was pipetted into a new plate and stored at −80 °C for long-term storage. For RT-qPCR, 5 µl RNA was amplified in a mix containing 1 µl β-actin, IFIT1, IFIT3, or OAS1 TaqMan primer/probe mix (Thermo Scientific), 5 µl TaqMan Fast Virus Master Mix (Life Technologies) and 9 µl dH2O. ISG expression levels were determined on a 7500 Real Time PCR System (Applied Biosystems) using a 1-step RT-qPCR program starting at a holding stage: 5 min at 50 °C; 20 s at 95 °C, and followed by 40 cycles of 3 s at 95 °C and 31 s at 60 °C. ISG fold induction were calculated using the comparative cycle threshold method (ΔΔCt) relative to the β-actin reference gene and normalized to untreated control samples. Fold increase of ISG expression was expressed as 2^-ΔΔCt.

### 2.6 HRSV replication kinetics in A549 cells

To assess replication kinetics of rHRSV^A11^EGFP (5) in WT and KO A549 cells, 150,000 cells were seeded in 24 well plates. The next day, KO and WT A549 cells were infected with rHRSV^A11^EGFP (5) at a multiplicity of infection (MOI) of 0.05 for 2 h. Supernatant (cell-free virus) and cells (virus dissemination and replication, determined as the percentage EGFP-positive cells) were obtained at 0.1, 1, 2, 3, and 4 DPI. For replication kinetics of rHRSV^A11^EGFP (5) in A549 cells (WT) following pretreatment with IFNs, 150,000 cells per well were seeded in 24 well plates. The following day, A549 cells were pretreated for 20 h with 5 ng/ml IFN-β, 50 ng/ml IFN-λ1, a combination of the two, or left untreated. Subsequently, A549 cells were washed and inoculated with rHRSV^A11^EGFP (5) at an MOI of 1 for 2 h. Supernatant (cell-free virus) and cells (virus dissemination and replication, determined as the percentage EGFP-positive cells) were obtained at 0.1, 1, 2, 3, and 4 DPI. Supernatant was diluted 1:1 in 50 % (w/v) sucrose and stored at −80 °C before titration in a 6-fold dilution series in quadruplicate on HEp2 cells. Virus concentrations were calculated using the Reed-Muench method and expressed in tissue culture infectious dose 50 % (TCID50) (Reed and Muench, 1938). Cells were transferred to a 96-well V-bottom plate. After centrifugation, pellets were washed in FACS buffer consisting of 2 mM EDTA (Sigma) and 0.05 % (v/v) BSA and fixed in 2 % (v/v) paraformaldehyde (PFA; Santa Cruz) prepared in PBS. Fixed cells were stored at 4 °C in the dark until flow cytometric analysis on a FACS Lyric (BD). Analysis was performed using FlowJo v10.8.1 (BD).

### 2.7 HRSV replication kinetics in AO at ALI

For growth kinetics in AO at ALI, cells were pretreated by adding 0.5 ml of 5 ng/ml IFN-β (2,5 ng/well) and/or 50 ng/ml IFN-λ1 (25 ng/well) in CBM to the basolateral medium for 20 h. After pretreatment, the basolateral compartment was washed once with DPBS++. The apical side was washed to remove excess mucus prior to HRSV inoculation. AO at ALI were inoculated with rHRSV^A11^EGFP (5) at an MOI of 0.05 in 50 µl CBM for 2 h and subsequently washed with 200 µl DPBS++ 3 times. The last wash was saved as an apical wash medium control at timepoint 2 HPI. Apical washes (cell-free virus) were obtained, and EGFP-positive cell surface area (virus dissemination and replication) were measured at 0.1, 1, 2, 3, 4, 5, 6, 7, 8, 11 and 14 DPI. Apical washes were obtained by adding 200 µl DPBS++ to the apical compartment, incubating for 20 min, and carefully pipetting up and down. Apical washes were diluted 1:1 in 50 % (w/v) sucrose and stored at −80 °C until use. Titrations of apical washes were performed as described before. For the analysis of the EGFP-positive surface area, fluorescent scans of the AO at ALI were made on a Typhoon™ biomolecular imager (Amersham). Fluorescent scans were analyzed using ImageJ (Fiji) to determine the percentage GFP-positive surface area (Schindelin et al., 2012).

### 2.8 Interferon detection in cell culture supernatant

IFN in supernatant of A549 cells and AO at ALI was quantified using the LEGENDplex™ human type 1/2/3 IFN panel (BioLegend) according to the manufacturer’s protocol. Reaction volume was modified, using 12,5 µL of sample and reagents instead of 25 µL. Beads were measured on a FACS Lyric (BD), and data was analyzed using LEGENDplex™ data analysis software suite (BioLegend; biolegend.com/legendplex).

### 2.9 Bacterial stocks

*S. pneumoniae* (ATCC strain 19F), *C. pseudodiphtheriticum* (clinical isolate), *H. influenzae* (ATCC, 9334) and *S. aureus* (ATCC, 29,213) were obtained as glycerol stocks, kindly provided by the department of Medical Microbiology and Infectious Diseases, Erasmus MC. Bacteria were cultured for 18–24 h on agar plates before use in experiment: *S. pneumoniae, C. pseudodiphtheriticum* and *S. aureus* were culture on blood agar plates (TSA) and *H. influenzae* was cultured on chocolate agar plates. Prior to experiment, bacteria were harvested in 5 mL F10F (Ham’s Nutrient Mixture F12 supplemented with 10 % FBS) without PSG and diluted to the preferred OD600 measured on spectrophotometer (Thermoscientific, Genesys 20) corresponding to a specific concentration of colony forming units (CFU)/ml (Table 1).

**Table 1:** Bacterial CFU counts at their specific OD600 concentration (*n* = 2).

**Bacterium**	**OD_600_ value**	**CFU/ml**
*S. pneumoniae*	0.01	3,1 × 10^6^
*C. pseudodiphtheriticum*	0.04	5,3 × 10^7^
*H. influenzae*	0.04	1,9 × 10^8^
*S. aureus*	0.001	8,8 × 10^6^

### 2.10 Bacterial co-culture experiments

A549 cells were seeded in 24-wells plates at 200,000 cells/ml F10F+PSG and incubated overnight at 37 °C + 5 % CO_2_. Chloramphenicol was added at 5 (*S. pneumoniae* and C. *pseudodiphteriticum*) or 10 (*H. influenzae* and *S. aureu*s) µg/ml. These bacteriostatic chloramphenicol concentrations were determined based on minimal inhibitory concentrations (MIC) assays. A549 cells were washed twice with PBS and incubated with bacterial suspensions for 24 h. Subsequently, A549 cells were washed twice and inoculated with rHRSV^A11^EGFP (5) at MOI of 3 for 2 h. Samples were taken at 24, 48 and 72 HPI, by harvesting the supernatant, and trypsinizing and fixing the cells. For co-cultures with AO at ALI, cells were washed twice apically with dPBS++ to remove mucus, after which 50 µl of the bacterial suspension with 5 or 10 µg/ml chloramphenicol was added apically; additionally, CBM with 5 or 10 µg/ml chloramphenicol was added basolaterally. Co-cultures were incubated for 16 h before the suspension was removed and stored at −80 °C for further analysis. After 8 h, AO at ALI were inoculated with rHRSV^A11^EGFP (5) at a MOI of 0.05 for 2 h. AO at ALI were washed and samples were taken at 1, 2, 3 and 4 DPI, including apical wash with dPBS++ for TCID_50_ calculations and removal of cells from transwells for fixation and readout by flow cytometry to determine EGFP positive cells. Co-culture of bacteria with Ap-O AO was performed by incubating differentiated Ap-O AO with the bacteria in Pneumacult apical-out airway organoid medium (without Heparin to enable HRSV infection) for 24 h. After 24 h, Ap-O AO were taken out of the 24 well plates and washed with a reversible cell strainer (37 µM; Stemcell Technologies). Ap-O AO were subsequently split over 96-well U bottom plates (±100 Ap-O AO per well) and inoculated with rHRSV^A11^EGFP (5). Supernatant was harvested at 1, 2 and 3 DPI, and Ap-O AO were fixed to determine percentage of HRSV-infected Ap-O AO on a CTL Immunospot.

### 2.11 Bacterial staining

Bacteria were stained with PKH26 staining (Sigma-Aldrich), a red, fluorescent cell membrane marker. Staining was performed according to the manufacturer’s instructions, with as only exemption that the PKH26 concentration was halved. After staining, the bacteria were added to A549 cells and co-cultured as described above. Plates were fixed at 24 h post HRSV infection and imaged on a LSM700 confocal microscope using ZEN software (Zeiss).

### 2.12 Viral interference assays

For the viral interference assays, HPIV-3 was inoculated at a low dose on washed AO at ALI for 2 h. Subsequently, AO at ALI were washed twice and the second wash was stored as 2 HPI timepoint. Samples were obtained daily and split for storage in lysis buffer or for detection of cytokines and titrations. Three days post HPIV-3 infection, AO at ALI were inoculated with rHRSV^A11^EGFP (5) at an MOI of 0.05 for 2 h. Subsequently, AO at ALI were washed twice and the last wash was stored. Additional samples were obtained at 1, 2, 3, 4, 5, 6, 7, 8, 11 and 14 DPI, and split for storage in lysis buffer, for the detection of cytokines, and for titrations (in 25 % sucrose). At 0, 2, 4, 6, 8 and 14 DPI, part of the supernatant was additionally used fresh to measure tissue damage by lactate dehydrogenase (LDH) assay (CytoTox 96® Non-Radioactive Cytotoxicity Assay Promega) according to the manufacturers protocol. An unrelated AO at ALI well was lysed as positive control to determine maximum amount of LDH release. In addition, at 0, 2, 4, 6, 8 and 14 DPI a single AO at ALI well per condition was fixed using 2 % paraformaldehyde (v/v) for 20 min and subsequently stored in PBS at 4 °C. Supernatant was titrated in a 6-fold dilution series in quadruplicate on HEp2 cells. After 3–5 days, plates were fixed and stained for HPIV-3 infection (with a parainfluenza virus type 3 polyclonal antibody (PA17235 Life Technologies; Life Technologies; 1:200) using a secondary donkey anti-goat IgG (Alexa Fluor 647, Life Technologies, 1:200). HRSV infection was detected based on EGFP and HPIV-3 infection based on AF647. Virus concentrations were calculated using the Reed-Muench method and expressed in tissue culture infectious dose 50 % (TCID50).

### 2.13 Indirect immunofluorescence staining

Transwell membranes were excised, washed twice, permeabilized with 0.2 % Triton-X (v/v), and blocked in 2 % normal goat serum (NGS, v/v) in DPBS for 30 min. Membranes were incubated with moAb for 60 min in staining buffer containing 2 % NGS and 2 % (w/vol) bovine serum albumin (BSA). Cilia were stained with anti-acetylated tubulin (clone 6–11B-1; Alexa Fluor 647; Santa Cruz Biotechnologies, 1:200), the HRSV entry receptor was stained with a CX3CR1 antibody (clone 2A9–1; Alexa Fluor 488, Life Technologies/Invitrogen, 1:20), HPIV-3 infected cells were stained with a parainfluenza virus type 3 polyclonal antibody (PA17235 Life Technologies; Life Technologies; 1:200) using a secondary donkey anti-goat IgG (Alexa Fluor 647, Life Technologies, 1:200). Hoechst was added during the last 10 min of the staining (Life Technologies/Invitrogen). Membranes were washed three times with staining buffer and mounted in Prolong antifade mounting medium (Life Technologies/Invitrogen). Membranes were imaged on a LSM700 confocal microscope using ZEN software (Zeiss) and analyzed in Fiji, including the calculation of co-localization of bacteria and HRSV-infected cells and percentage CX3CR1 expression on control and HPIV-3 infected AO at ALI (Schindelin et al., 2012).

### 2.14 Histology

Fixed transwells were excised, stored in formalin, and subsequently embedded in paraffin. Thin sections (3 μm) were prepared and stained with hematoxylin and eosin.

### 2.15 Statistical analyses

Statistical analyses were performed on datasets consisting of three technical replicates per experiment, with each experiment independently repeated twice. For AO at ALI experiments, two donors were used (each in technical triplicate and repeated twice). Data are presented as mean ± SEM, with the number of replicates indicated in the figure legends. To detect differences between conditions at different timepoints compared to the mock or WT A549 cells a 2-way ANOVA was performed with multiple comparisons. Growth curves of HPIV-3 + HRSV, HPIV-3 only, HRSV only or control AO at ALI were analyzed using a two-way mixed-effects model (REML) with time as a repeated measure and group as a fixed factor. Post-hoc multiple comparisons were conducted using Tukey correction to compare groups at each time point. Model assumptions were checked by inspecting residual plots, QQ plots, and homoscedasticity plots. A P-value <0.05 was considered statistically significant, with P-values being annotated as NS (*P* > 0.05), * (*P* < 0.05), ** (*P* < 0.01), *** (*P* < 0.001), or **** (*P* < 0.0001). Statistical analyses were performed using GraphPad Prism 10, results are shown in the figures and/or in Supplemental Tables 1-8.

## 3 Results

### 3.1 Enhanced HRSV infection in IFNAR1 and IFNLR1 knockout cells

**Fig. 1:**
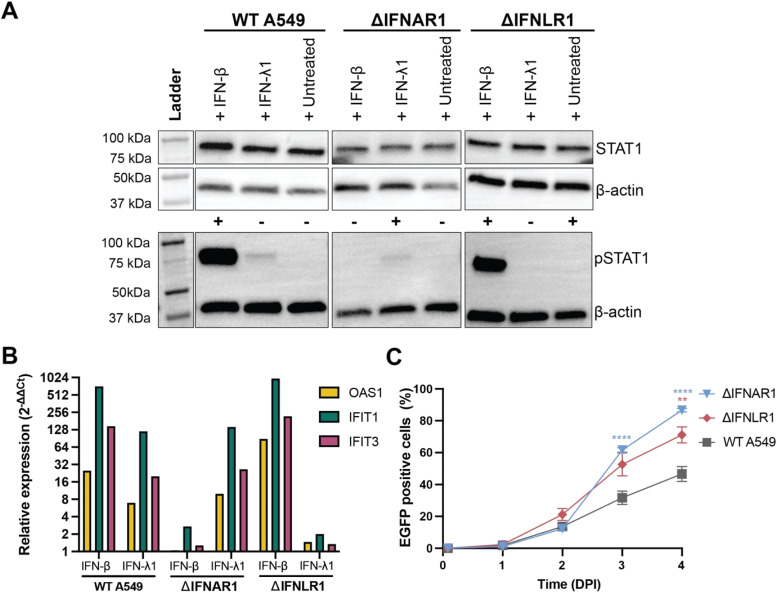
**HRSV permissiveness of IFN-deficient A549 cells.** (A) Detection of STAT1 and pSTAT1 in untreated and IFN treated WT and KO cells by Western blot; β-actin was used as an internal loading control. + indicates the presence of a band for pSTAT1, - the absence of a band for pSTAT1. (B) Detection of ISG expression (OAS1, IFIT3 and IFIT1) in untreated and IFN treated WT and KO cells by RT-PCR and calculated relative to untreated cells. One representative experiment is shown. (C) HRSV dissemination in untreated and IFN treated WT and KO cells quantified as percentage of EGFP positive cells as determined by flow cytometry. Differences between KO cells and WT cells were tested by two-way ANOVA with multiple comparisons (* = *P* < 0.05, ** = *P* < 0.01, *** = *P* < 0.001, **** = *P* < 0.0001). Data is represented as mean ± SEM of technical replicates from at least two independent experiments. Abbreviations: A549 = human lung adenocarcinoma cell line; WT = wildtype; IFN = interferon; IFNAR1 = interferon alpha/beta receptor 1; IFNLR1 = interferon lambda receptor 1; STAT1 = signal transducer and activator of transcription 1; pSTAT1 = phosphorylated signal transducer and activator of transcription 1; kDa = kilodalton; OAS1 = 2′−5′-oligoadenylate synthetase 1; IFIT1 = interferon-induced protein with tetratricopeptide repeats 1; IFIT3 = interferon-induced protein with tetratricopeptide repeats 3; EGFP = enhanced green fluorescent protein; DPI = days post infection.

A549 knockout cells lacking expression of IFNAR1 or IFNLR1 were treated with IFN-β or IFN-λ1, to confirm the disruption of the IFN signalling pathway. In WT A549 cells IFN treatment resulted in the phosphorylation of STAT1, demonstrating functionality of both the type I and III IFN pathway (Fig. 1A). A549 cells deficient for IFNAR (∆IFNAR1) could not phosphorylate STAT1 after treatment with IFN-β, but IFN-λ1 treatment led to the formation of pSTAT1, indicating the functional knockout of the type I IFN pathway while maintaining a functional type III IFN pathway. *Vice versa*, A549 cells deficient for IFNLR (∆IFNLR1) showed phosphorylation of STAT1 after IFN-β but not IFN-λ1 treatment. In addition to detection of pSTAT1, the phenotype was confirmed by lack of induction of three different ISGs (OAS1, IFIT3 and IFIT1) in the respective KO cells after IFN treatment (Fig. 1B).

Next, WT A549, and ∆IFNAR1 and ∆IFNLR1 cells were infected with HRSV, and infection percentages were measured over time. In WT A549 cells, HRSV infection reached an infection percentage of approximately 47 % at 4 DPI (Fig. 1C). Notably, infection percentages at 4 DPI were significantly higher in ∆IFNAR1 (87 %) and ∆IFNLR1 (71 %) cells. Differences in cell-free virus loads in the supernatants were less pronounced, although a significant increase was observed in supernatants of HRSV-infected ΔIFNAR1 cells (**Supplemental Figure 1A**).

### 3.2 IFN pretreatment of A549 cells and AO at ALI suppresses HRSV replication

**Fig. 2:**
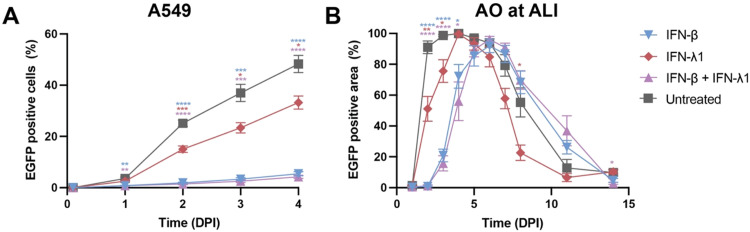
**Type I and III IFN pretreatment reduced HRSV dissemination in A549 cells and AO at ALI.** (A) HRSV dissemination in untreated and IFN pretreated A549 cells. HRSV dissemination was quantified as percentage of EGFP positive cells as determined by flow cytometry. Data are based on two independent experiments; each performed in triplicate. (B) HRSV dissemination in untreated and IFN pretreated AO at ALI. HRSV dissemination was quantified as percentage of EGFP positive surface area as determined by taking fluorescent images. Data represents two independent experiments, with 2 different organoid donors, performed in triplicate. Differences between pretreatment conditions and untreated A549 cells were tested by two-way ANOVA with multiple comparisons (* = *P* < 0.05, ** = *P* < 0.01, *** = *P* < 0.001, **** = *P* < 0.0001). Mean ±SEM is shown. Abbreviations: EGFP = enhanced green fluorescent protein; DPI = days post infection; IFN = interferon.

Next, we evaluated the effect of pretreatment with IFN on HRSV replication. WT A549 cells were pretreated with IFN-β, IFN-λ1, or a combination of the two. Pretreatment with IFN-λ1 resulted in a modest reduction of HRSV infection at 4 DPI (48 % of untreated A549 cells *vs.* 33 % IFN-λ1 treated cells; Fig. 2A). IFN-β exerted a stronger antiviral effect, resulting in significantly reduced HRSV replication (5 % at 4 DPI). The combination treatment did not result in an additional effect (4 % at 4 DPI). When measuring cell-free virus, a significant reduction was observed in the supernatant of A549 cells pretreated with IFN-β alone, or in combination with IFN-λ1 (**Supplemental figure 1B**).

Next, we pretreated ciliated airway epithelial cells (AO at ALI) and followed HRSV infection for 14 days. Like A549 cells, IFN-λ1 pretreatment conferred a modest reduction in HRSV infection (99 % infected area of untreated cells *vs* 76 % of IFN-λ1 pretreated cells at 3 DPI) and IFN-β exerted a stronger inhibitory effect (21 % infected area at 3 DPI) (Fig. 2B). Despite these antiviral effects, HRSV continued to spread; in all conditions AO at ALI became fully infected at later time points. Interestingly, IFN-λ1 pretreatment led to an accelerated clearance of HRSV infection compared to untreated AO at ALI, although only significant at 8 DPI. The measurement of cell-free virus supported these findings with less pronounced differences (**Supplemental figure 1B**). These results demonstrate that the presence of type I and type III interferons prior to HRSV exposure reduces infection, primarily reflected by a decrease in infected cells rather than in cell‑free virus, with IFN-β having a pronounced effect and IFN-λ a modest one.

### 3.3 Bacterial exposure alters HRSV infection dynamics

**Fig. 3:**
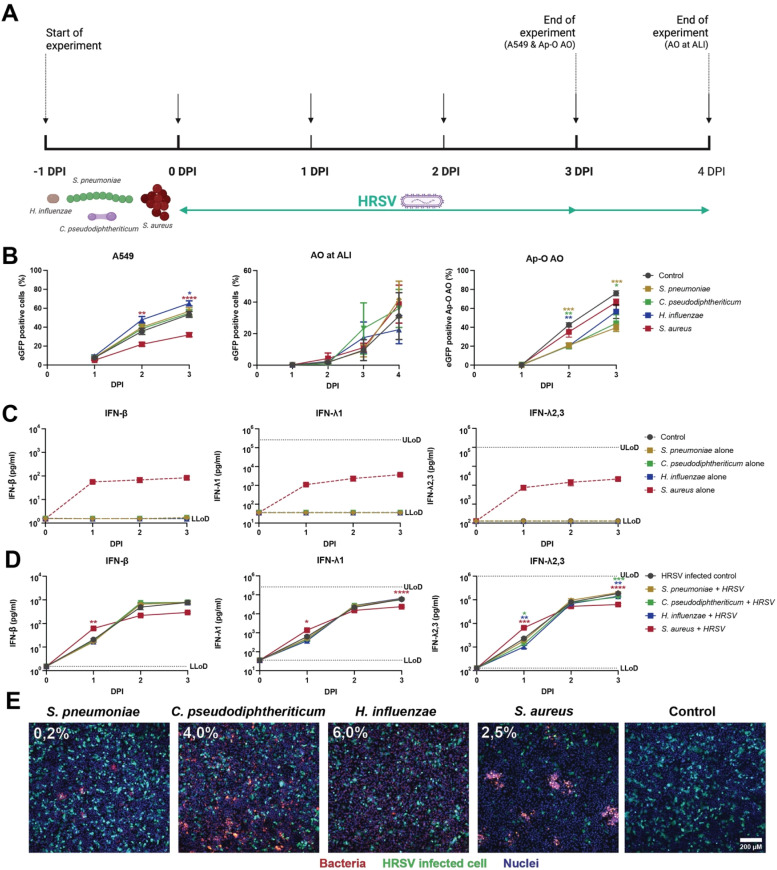
**Bacterial co-culture affects HRSV infection and dissemination.** (A) Timeline of bacterial co-culture experiments. Black arrows indicate timepoints at which supernatant was collected for cytokine detection and HRSV-infected cells were measured/imaged. (B) HRSV dissemination in A549 (left), AO at ALI (middle) and Ap-O AO (right) in the absence and presence of bacterial co-culture. HRSV dissemination was quantified as percentage of EGFP positive cells/Ap-O AO. Data represents at least two independent experiments, performed in triplicate. (C, D) Induction of IFN responses by bacteria alone (C) or bacterial co-culture with HRSV infection (D) on A549 cells. Differences between conditions and control without bacteria were tested by two-way ANOVA with multiple comparisons (* = *P* < 0.05, ** = *P* < 0.01, *** = *P* < 0.001, **** = *P* < 0.0001). Mean ±SEM are shown. (E) Confocal images of A549 cells co-cultured with PKH-26 labelled bacteria (red) 24 h after HRSV infection (green). Nuclei are stained in blue (Hoechst). The co-localization percentages are displayed in the upper left corner. Abbreviations: A549 = human lung adenocarcinoma cell line; AO at ALI = airway organoids at air-liquid interface; Ap-O AO = apical-out airway organoid; EGFP = enhanced green fluorescent protein; DPI = days post infection; IFN = interferon; ULoD = upper limit of detection; LLoD = lower limit of detection.

To determine whether the resident microbiome modulates HRSV replication, we next assessed HRSV infection dynamics after exposure of cells to four different bacterial species: *S. pneumoniae, C. pseudodiphtheriticum, H. influenzae* and *S. aureus* (Fig. 3A). To generate culture conditions where cells could be exposed to bacteria without causing cell death, we added predefined optimized bacteriostatic concentrations of chloramphenicol. Co-culturing A549 cells with *H. influenzae* resulted in a modest increase in HRSV infection percentages compared to the virus-only control at 2 and 3DPI, with approximately 12 % more infected cells at 3 DPI (Fig. 3B). In contrast, co-culture with *S. aureus* significantly inhibited HRSV infection and dissemination, with 32 % infected cells of the *S. aureus* co-cultures, compared to 54 % for control cultures at 3 DPI. Co-culture with *S. pneumoniae* or *C. pseudodiphtheriticum* did not affect HRSV infection percentages. To further assess these interactions in physiologically relevant models, we performed co-culture experiments on AO at ALI and Ap-O AO. In AO at ALI, no significant effects of co-culture with bacteria on HRSV infection and dissemination were observed (Fig. 3B). Interestingly, in experiments performed on Ap-O AO, a significant decrease of HRSV infection and dissemination was observed in co-cultures with *S. pneumoniae* or *C. pseudodiphtheriticum* at 3 DPI, in contrast to results obtained on A549 cells (Fig. 3B)*.*

### 3.4 Bacterial influence on IFN production and co-localization of bacteria with HRSV-infected cells

To assess whether the altered HRSV infection dynamics correlated with the production of type I and III IFN in response to bacterial exposure, IFN concentrations were measured in supernatants. Notably, exposure to most bacteria in the absence of HRSV did not induce production of IFN. However, *S. aureus* exposure of A549 cells resulted in a marked increase in IFN-β and IFN-λ production (Fig. 3C). Interestingly, while HRSV infection induced IFN-β and IFN-λ expression by itself, co-culture with of A549 cells with *H. influenzae* in the presence of HRSV infection slightly reduced IFN-λ2,3 responses at 1 and 3 DPI (Fig. 3D). This could (partially) explain the observed increased HRSV replication. IFN responses in AO at ALI or Ap-O AO co-cultured with bacteria were not clearly distinguishable or below detection limits, respectively (**Supplemental figure 2**).

Since bacterial presence can modulate HRSV infection and induced interferon responses, we next assessed whether bacteria directly interacted with HRSV-infected cells. Labeled bacteria were co-cultured with A549 cells for 24 h, followed by HRSV infection for an additional 24 h. Non-adherent bacteria were removed by washing. Fluorescent imaging of bacteria and HRSV-infected cells revealed distinct patterns across the bacterial species (Fig. 3E). *S. pneumoniae* and *S. aureus* formed aggregates in the culture, while *C. pseudodiphtheriticum* and *H. influenzae* were evenly dispersed. Similar as observed before, HRSV infection and dissemination was reduced in A549 cultures co-incubated with *S. aureus*. The highest colocalization between HRSV-infected cells and bacteria was observed for *H. influenzae* (6.0 %), the lowest for *S. pneumoniae* (0.2 %)*.*

### 3.5 Prior viral infection affects subsequent HRSV infection

**Fig. 4:**
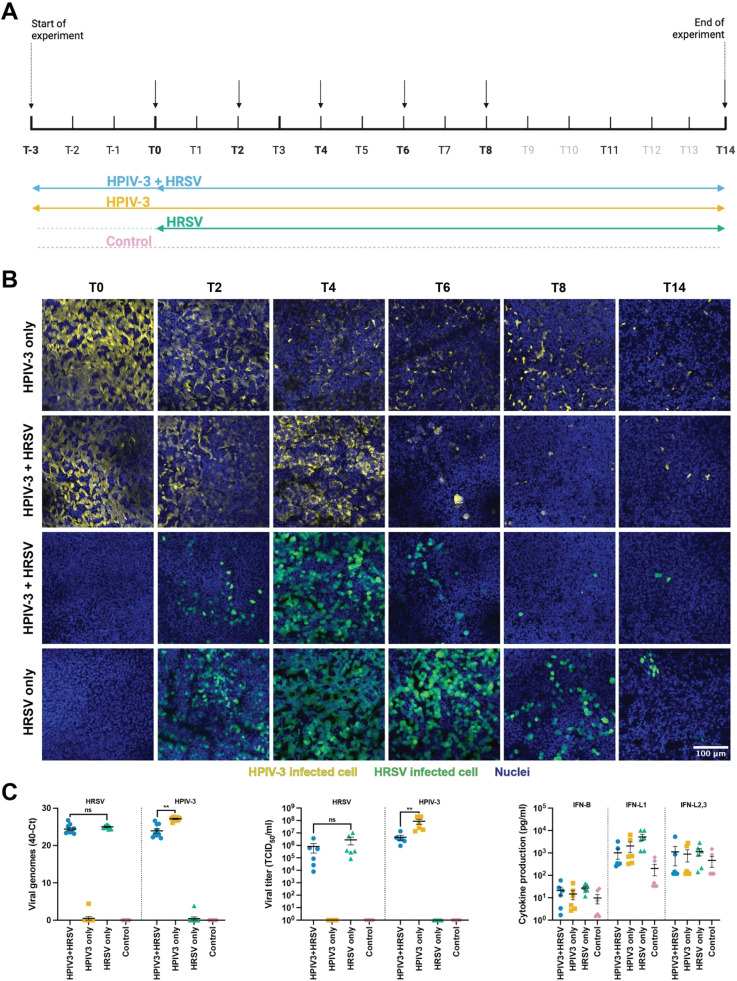
**Prior HPIV-3 infection affects the outcome of subsequent HRSV inoculation.** (A) Timeline of viral interference experiment. Different conditions are indicated by the colored arrows. Black arrows indicate timepoints on which a transwell was fixed for immunofluorescent staining and LDH assays were performed. (B) Immunofluorescent staining of fixed transwells. HPIV-3 infection is shown in yellow, HRSV infection in green, and nuclei (hoechst) in blue. The rows annotated with ‘HPIV3 + HRSV’ are the same well, fluorescent channels were separated. Representative images are shown. (C) Viral genomes, determined by qPCR, viral titers, determined by TCID_50_ titrations, and cytokine responses, measured via LEGENDplex in samples obtained at T6. Data represents two independent experiments, with 2 different organoid donors. Mean ±SEM is shown. Statistical significance was assessed using a two-way mixed-effects model (REML) followed by Tukey’s multiple comparisons test at each time point. Only the statistics of relevance are shown. Full dynamics are shown in **Supplemental figure 3**. Abbreviations: *T* = timepoint, HPIV-3: human parainfluenza virus 3, HRSV = human respiratory syncytial virus; TCID_50_/ml = 50 % tissue culture infectious dose per milliliter.

Next, we investigated whether prior viral exposure altered subsequent HRSV replication dynamics. To this end, AO at ALI were infected with HPIV-3 and subsequently inoculated with HRSV. Cultures infected with either HPIV-3 or HRSV served as controls (Fig. 4A). At 3 DPI with HPIV-3 (T0, prior to HRSV infection), HPIV-3-infected cells were readily detected in AO at ALI (Fig. 4B). Notably, in wells subsequently inoculated with HRSV, HPIV-3-infected cells were cleared more rapidly compared to wells not inoculated with HRSV. This was particularly evident at 6 and 8 days post HRSV infection (T6 and T8). At the same time, HRSV-infected cells were cleared more rapidly in wells co-infected with HPIV-3, indicating reciprocal interference between the two viruses. These effects were confirmed by viral load and titer measurements, albeit less pronounced (Fig. 4C for T6, **Supplemental figure 3** for the replication dynamics). At T6, taken as representative timepoint between T0 and T14, higher IFN-λ1 levels were detected in the HRSV-only condition compared to HPIV-3 or a combination of HPIV-3 and HRSV, although not significant. No differences were observed in the production of IFN-β and IFN-λ2,3.

### 3.6 Host factors underlying viral interference

**Fig. 5:**
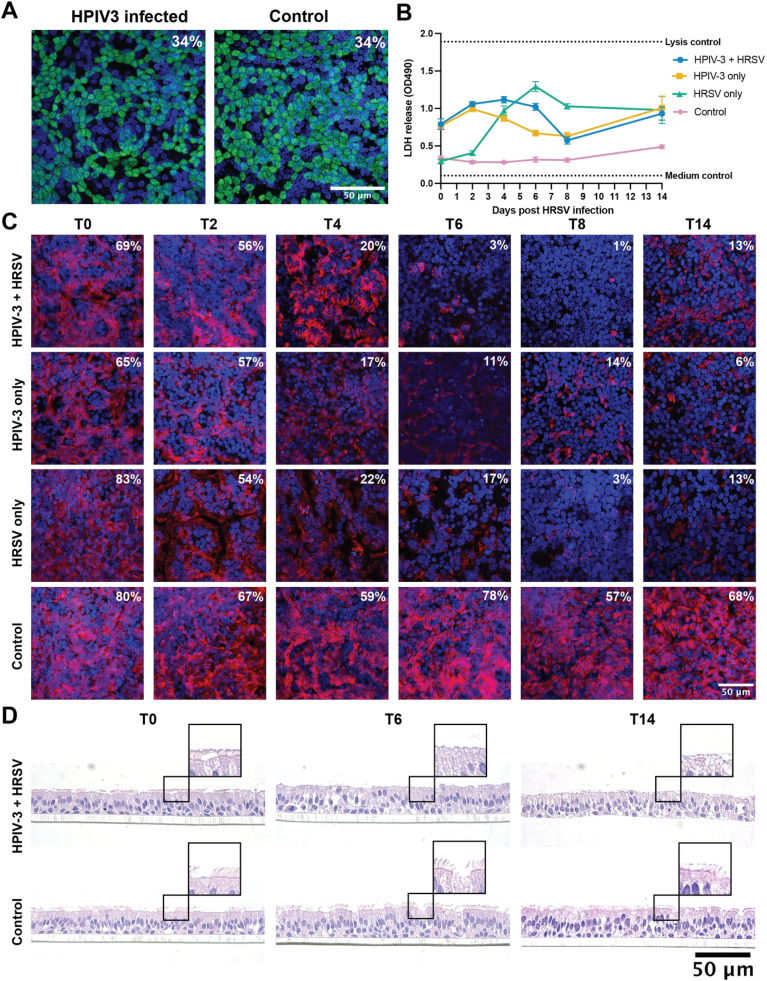
**Host factors underlying viral interference.** (A) CX3CR1 receptor expression (green) stained on transwells fixed at T0. The percentage CX3CR1‑positive area was quantified from ten images acquired across the entire well, with the average measured region indicated in white in the upper‑right panel. (B) Cell death assessed by LDH release assay. Dotted lines indicate lysis control or negative controls. Data represents two independent experiments, with 2 different organoid donors. Mean ±SEM is shown. Statistical significance was assessed using a two-way mixed-effects model (REML) followed by Tukey’s multiple comparisons test at each time point. Statistics are depicted in **Supplemental Table 2**. (C) Presence of cilia on AO at ALI (red) as determined by fluorescent staining at different timepoints. The percentage cilia‑positive area was quantified from five images acquired across the entire well, with the average measured region indicated in white in the upper‑right panel. (D) Sidecuts of transwell filters to determine whether cilia loss reflected changes to pseudostratified epithelium. Slides were stained with hematoxylin & eosin (H&E). All H&E slides over all timepoints and conditions are shown in **Supplemental figure 4**. Abbreviations: *T* = timepoint, HPIV-3: human parainfluenza virus 3, HRSV = human respiratory syncytial virus; LDH = lactate dehydrogenase; OD490 = Optical Density measured at a wavelength of 490 nanometers.

To elucidate the mechanism underlying the observed viral interference, we investigated whether HPIV-3 infection alters host factors critical for HRSV entry. First, we assessed the effect of HPIV-3 infection on the expression of CX3CR1, the putative HRSV receptor expressed by ciliated epithelial cells. CX3CR1 expression was unchanged between conditions, as AO at ALI pre-infected with HPIV-3 showed only a marginal reduction of receptor expression compared to uninfected wells (34,41 % ±0,56 vs 34,00 % ±0,93 of surface area), with most epithelial cells retaining receptor expression (Fig. 5A). Next, we performed LDH assays to evaluate if tissue damage contributes to the observed slower increase and faster decline of HRSV infection in the presence of HPIV-3 infection. LDH was most abundant in co-infected AO at ALI, although HPIV-3 only and HRSV only conditions reached similar levels by T1 or T6, respectively (Fig. 5B). Given the increase in LDH indicates loss of cell membrane integrity, we additionally assessed the ciliary structure by acetylated α-tubulin staining. Cilia were almost completely lost in all infection conditions by T8 in HRSV- and or HPIV-3-infected conditions, although mostly apparently in HRSV-infected cultures, with partial restoration by T14 (Fig. 5C). To determine whether cilia staining loss was caused by damage to the cilia themselves, or rather reflected epithelial cell depletion from the cultures, transwell filters were embedded in agarose, sectioned, and stained (Fig. 5D, **Supplemental figure 4**). Histological analyses showed that epithelial integrity was mostly maintained, as the layers had a consistent thickness, but the cilia themselves were shortened or absent. By T14, cilia regeneration was evident.

## 4 Discussion

IFN responses can modulate HRSV infection dynamics *in vitro* and *in vivo*, and specific bacteria or viruses correlate with differences in disease severity. Here, we investigated whether exposure to these microbes alters HRSV infection dynamics in human airway epithelial cells and whether such effects are mediated by IFN-driven mechanisms. Our findings confirm that IFN-β exerts significantly stronger antiviral activity than IFN-λ1, while IFN-λ1 seems to have a slower but longer lasting antiviral effect. We observed that several bacterial species can modulate HRSV infection, but in a culture model-dependent manner. Prior HPIV-3 infection of AO at ALI markedly shortened the course of subsequent HRSV infection, accompanied by present interferon responses and noticeable cilia shortening, highlighting how subsequent viral infections can reshape epithelial architecture and influence pathogen susceptibility and interactions.

Our observation that IFN-β was significantly more potent than IFN-λ1 in inhibiting HRSV infection is consistent with the fundamental difference in their signaling dynamics. Type I IFNs induce a rapid and robust ISG response that peaks within hours and acts systemically, creating a strong but transient antiviral state (Lazear et al., 2019). This explains the pronounced increase in HRSV infection in A549 cells lacking the type I IFNs signaling pathway (∆IFNAR1) and the evident reduction in HRSV replication following IFN-β pretreatment in both A549 cells and AO at ALI. In contrast, type III IFNs act primarily at epithelial surfaces and elicit a slower, more moderate, ISG induction, resulting in a prolonged but less intense antiviral effect. While this sustained response may be advantageous for limiting inflammation at mucosal barriers, it appears insufficient to match the immediate antiviral pressure elicited by IFN-β in our experimental setting. Our observations are consistent with previous work employing IFNAR and IFNLR knockout cell lines and interferon pretreatment in the context of HRSV infection (Czerkies et al., 2022). Other studies utilized knockout mouse models lacking receptors for type I, type III, or both IFNs. The mice lacking only IFNAR were more susceptible to HRSV than those with deleted IFNLR, whereas double receptor knockout mice showed the highest viral loads (Mordstein et al., 2010). Interestingly, reduced viral spread did not always correspond to reduced viral titers, emphasizing that antiviral effects may limit dissemination more than virus shedding. This distinction is relevant when interpreting the differential effects of type I and III IFNs. Together, these findings highlight how the kinetics and tissue-specific properties of IFN classes shape antiviral efficacy.

Different members of the airway microbiome have been linked to distinct community-state profiles associated with mild or severe HRSV-associated disease (Duttweiler et al., 2004; Thorburn et al., 2006; Thorburn and Van Saene, 2007; O'Brien et al., 2009; Watt et al., 2009; Tomosada et al., 2013; Yoshida et al., 2013; Nguyen et al., 2015; de Steenhuijsen Piters et al., 2016; Kanmani et al., 2017; Man et al., 2019; Zhang et al., 2020). Profiles dominated by respiratory pathogens such as *S. pneumoniae, H. influenzae*, and *M. catarrhalis* correlate with severe bronchiolitis, whereas commensal-rich profiles (*Dolosigranulum, Corynebacterium, Lactobacillus*) correlate with milder disease and stronger antiviral responses. Our findings show that bacteria can modulate HRSV infection *in vitro* in a species- and model-dependent manner. In A549 cells, *H. influenzae* enhanced HRSV infection, potentially through suppression of antiviral signaling. Conversely, *S. aureus* induced IFN even without viral infection, which may explain its protective effect by pre-activating epithelial antiviral pathways. However, these patterns were not reproduced in airway organoids, where *S. pneumoniae* and *C. pseudodiphtheriticum* instead appeared to limit HRSV infection.

The complex interplay between bacterial signals and HRSV is highlighted in other studies where prior bacterial exposure exacerbated disease. As an example, intranasal priming with LPS or *A. baumannii* before HRSV infection in mice increased lung inflammation via IL‑1α and TNF‑α (Owen et al., 2024). Clinical relevance is underscored by planned controlled human infection model studies assessing whether pneumococcal colonization increases HRSV susceptibility (Brito-Mutunayagam et al., 2025). Certain commensals such as *Lactobacillus rhamnosus* reduce HRSV replication and preserve epithelial integrity through enhanced IFN-β responses (Yarlagadda et al., 2025). The interplay between bacterial signals and HRSV is bidirectional, as the HRSV glycoprotein G can bind *H. influenzae* and *S. pneumoniae*, promoting bacterial adherence and co-colonization (Avadhanula et al., 2007). Collectively, these findings highlight that bacterial presence, pathogenic or probiotic, can profoundly shape HRSV outcomes by modulating inflammation, antiviral signaling, and epithelial barrier function. Differences between our *in vitro* models likely reflect the greater complexity of organoid systems, including mucus production, cilia movement, cellular diversity, and baseline immune status, which buffer or redirect microbial influences. In AO at ALI, the presence of goblet cell-derived mucus together with ciliary activity likely promotes bacterial clearance, consistent with our observation of bacteria accumulating at the transwell periphery. In contrast, Ap‑O AO lack goblet cells and mucus, allowing more direct bacterial-epithelial contact. Notably, this still resulted in a response distinct from that observed in A549 cells.

The disparity in results obtained when using A549 cells or airway organoids suggests that we have not yet identified an optimal model for studying bacterial co-cultures in the context of viral infection. Several studies have explored bacterial-epithelial co-culture systems using diverse strategies to mimic host-microbe interactions. However, most of these efforts have focused on the gastrointestinal tract rather than the respiratory system. For example, microfluidic platforms have been used to co-culture epithelial cells with commensal and pathogenic bacteria under controlled conditions, enabling real-time observation of competitive dynamics (Kim et al., 2010). More advanced models include organoid-based co-cultures where bacteria are microinjected into intestinal organoids to study localized interactions at mucosal surfaces (Puschhof et al., 2021). These studies collectively demonstrate that while co-culture models can reveal important mechanisms, they vary widely in complexity and physiological relevance, and no single system seems to fully capture the intricate interplay of the microbiome, epithelial barriers, immune priming and viral infection as observed *in vivo*.

Since interferon responses triggered by bacteria do not fully explain differences in HRSV-associated disease severity, other determinants are likely involved. We explored whether preceding viral infections could influence susceptibility and outcomes of HRSV infection and found that prior HPIV-3 infection of AO at ALI shortened the duration of subsequent HRSV infection. Although not the primary focus of this study, we also observed that HRSV infection reduced HPIV‑3 replication. The shortened HRSV infection duration did not seem to be explained by the high expression of IFN-λ in response to HPIV-3 infection. We therefore explored whether the effect could be attributed to other factors affecting host cells susceptibility. Whereas HPIV-3 infection did not influence the expression of the putative HRSV receptor CX3CR1, cultures infected with both viruses exhibited more rapid cilia loss compared to cultures infected with either virus alone. By day 14, cilia reappeared, suggesting the initiation of epithelial layer regeneration. Tissue damage was corroborated by the detection of an increase in LDH release in cultures infected with HPIV-3 and HRSV. Our findings underscore the complexity of viral co-infection scenarios, where prior viral exposure may modulate susceptibility to or severity of subsequent infections.

Although multiple studies investigated influenza A virus infection hampering subsequent HRSV infection epidemiologically, *in vitro*, and *in vivo* (Drori et al., 2020; Piret and Boivin, 2022; Kramer et al., 2024; Gilbert-Girard et al., 2025), viral interference studies with other respiratory viruses are lagging. Interestingly, mathematical modeling of respiratory viral coinfections suggests that the outcome of sequential infections is more strongly influenced by viral growth kinetics than immune mechanisms alone (Pinky and Dobrovolny, 2016). Their simulations predicted that the virus with the faster replication rate typically dominates, suppressing the slower-growing virus. Based on our qPCR and titration data, HPIV-3 seems to replicate at similar rates as HRSV. However, this theoretical framework implies that if HPIV-3 replicates more slowly than HRSV, preceding HPIV-3 infection may exert limited interference on HRSV, whereas the reverse scenario could favor HRSV suppression. These insights highlight the importance of timing and replication dynamics in shaping interactions between respiratory viruses.

Bacterial or viral respiratory infections rarely occur alone, and our results emphasize the complexity of interactions between host, viruses, and bacteria within the airway environment. We observed that prior HPIV-3 infection can accelerate clearance of subsequent HRSV infection and found a species-specific effect of bacterial co-exposure. This illustrates how sequential and concurrent infections or commensal presence reshapes epithelial susceptibility and immune status. These interactions have important clinical implications, as they could influence HRSV-associated disease severity, alter treatment efficacy, and complicate vaccine strategies by modulating IFN responses and tissue integrity. Our study is an important step towards establishing a robust bacterial–viral and viral-viral co-culture model. Future work should build on this foundation by developing more advanced systems, such as microfluidic platforms that integrate epithelial cells, microbiome members, and immune components, to better mimic the airway microenvironment.

## Funding

RDdV and LLAvD received funding from HERA EU4Health project DURABLE.

## Acknowledgements

The authors would like to acknowledge Chinmoy Saha, Astrid Heikema and Corné P. de Vogel, who supplied the bacterial species and offered their support in bacterial culture. In addition we want to thank Peter R. van Run and Debby Schipper for their work in embedding the transwells, sectioning them, and performing H&E staining.

## CRediT authorship contribution statement

**Laura L.A. van Dijk:** Writing – original draft, Visualization, Validation, Project administration, Methodology, Investigation, Formal analysis, Data curation. **Laurine C. Rijsbergen:** Writing – review & editing, Methodology, Investigation. **Alexander C. Havelaar:** Writing – review & editing, Investigation. **Yvette den Hartog:** Writing – review & editing, Investigation. **Rosanne W. Koutstaal:** Writing – review & editing, Investigation. **Kevin Groen:** Writing – review & editing, Investigation. **Rory D. de Vries:** Writing – review & editing, Supervision, Resources, Conceptualization. **Rik L. de Swart:** Writing – review & editing, Supervision, Resources, Conceptualization.

## Declaration of competing interest

The authors declare that the research was conducted in the absence of any relationships that could be considered as potential conflict of interest.

## Data Availability

Data will be made available on request.

## Appendix Supplementary materials

Image, application 1
