# Supplementary material for: Innate airway responses shape permissiveness to human respiratory syncytial virus
Source: Virus Res. 2026 Apr 27;368:199734. doi: 10.1016/j.virusres.2026.199734 (PMC13153649; doi:10.1016/j.virusres.2026.199734)
Supplement: Supplementary file 1 [file mmc1.pdf]

Supplemental Figure 1

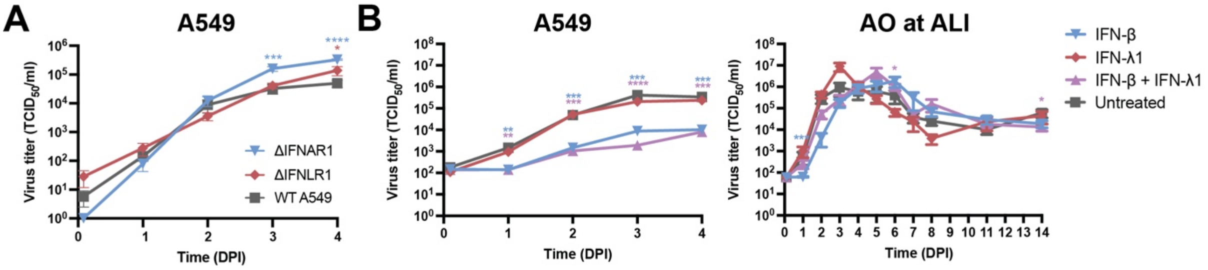

HRSV permissiveness in IFN-deficient A549 cells and after IFN pretreatment. (A) Production of cell-free HRSV by untreated and IFN treated WT and KO cells. (B) Production of cell-free HRSV in untreated and IFN pretreated A549 cells (left) or AO at ALI (right). Differences between KO cells and WT cells or IFN pretreatment and untreated cells were tested by two-way ANOVA with multiple comparisons (* = P<0.05, ** = P<0.01, *** = P<0.001, **** = P<0.0001). Data is represented as mean ± SEM of technical replicates from at least two independent experiments. For AO at ALI, data represents two independent experiments, with 2 different organoid donors, performed in triplicate * = P<0.05, ** = P<0.01, *** = P<0.001, **** = P<0.0001 compared to WT or untreated cells. Abbreviations: $TCID_{50}$/ml = 50% tissue culture infectious dose per milliliter; IFNAR1 = interferon alpha/beta receptor 1; IFNLR1 = interferon lambda receptor 1; WT = wildtype; AO at ALI: airway organoids at air-liquid interface; EGFP = enhanced green fluorescent protein; DPI = days post infection; IFN = interferon.

Supplemental Figure 2

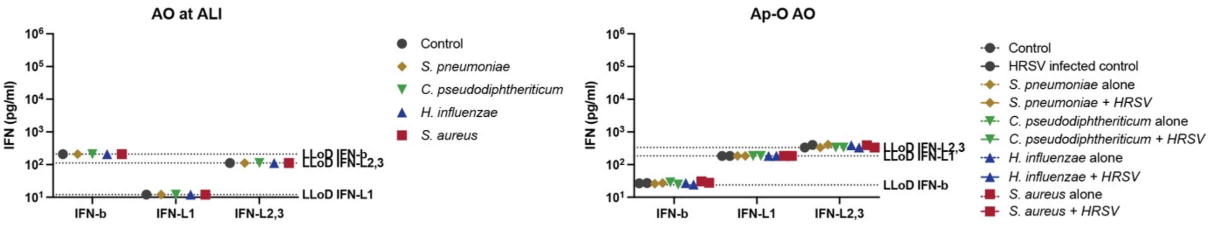

Bacterial co-culture does not induce IFN in AO. (A) Induction of IFN responses by bacteria in AO at ALI before start of HRSV infection. (B) Induction of IFN responses in Ap-O AO by bacteria alone or bacterial co-culture with HRSV infection at 3 DPI. One representative experiment is shown. Mean ±SEM are shown.

Supplemental Figure 3

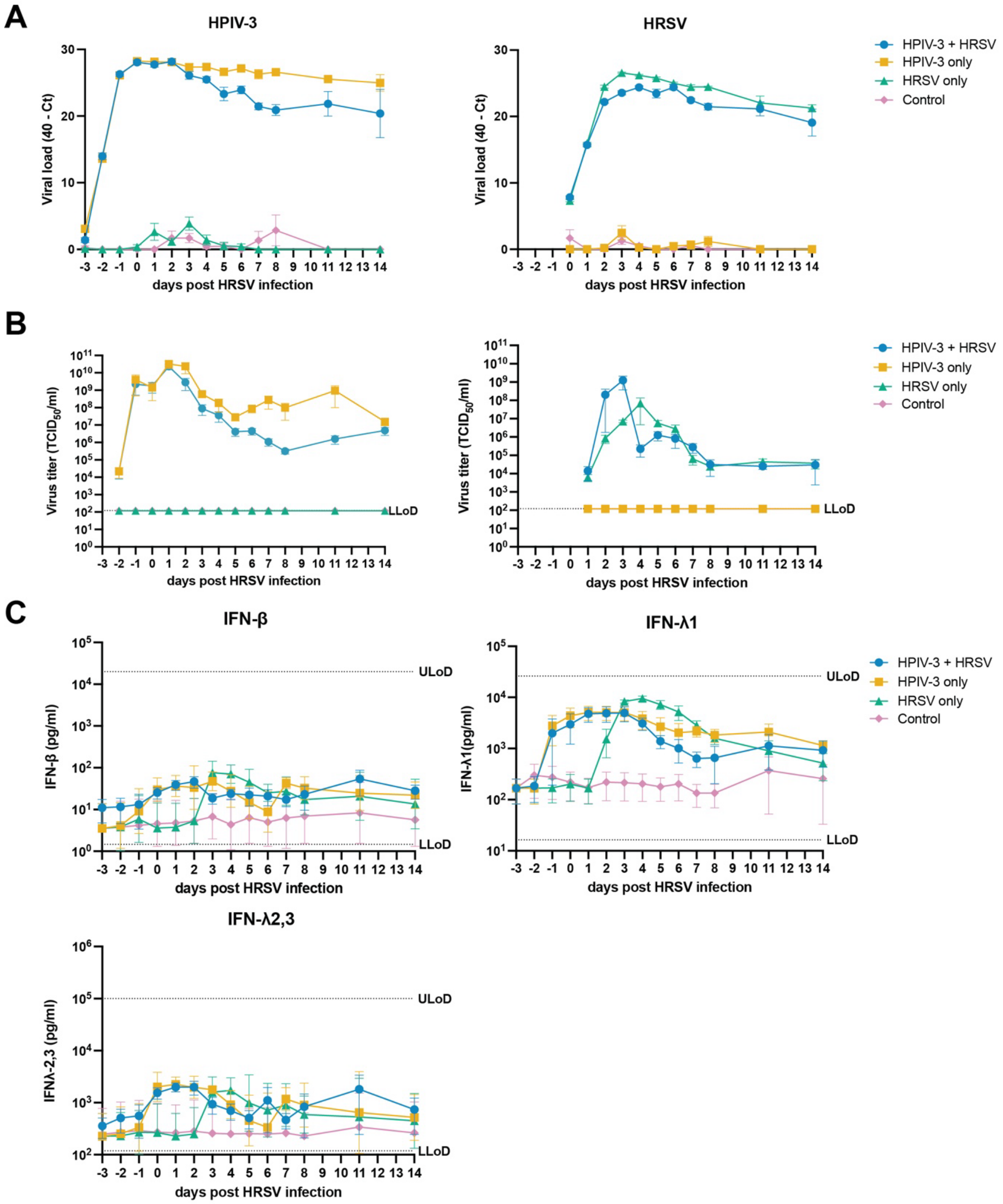

Impact of HPIV3 pre-infection on subsequent HRSV infection. (A) Quantification of viral genomes by qPCR in apical washes collected from AO at ALI. (B) Viral titers determined by titrations of the supernatant. (C) Cytokine responses assessed using LEGENDplex. Data represents two independent experiments, with 2 different organoid donors. Mean ±SEM is shown. Statistics are shown in **Supplemental table 2-8.** Abbreviations: HPIV-3: human parainfluenza virus 3, HRSV = human respiratory syncytial virus; $TCID_{50}$/ml = 50% tissue culture infectious dose per milliliter. ULoD = upper limit of detection; LLoD = lower limit of detection; IFN = interferon.

Supplemental Figure 4

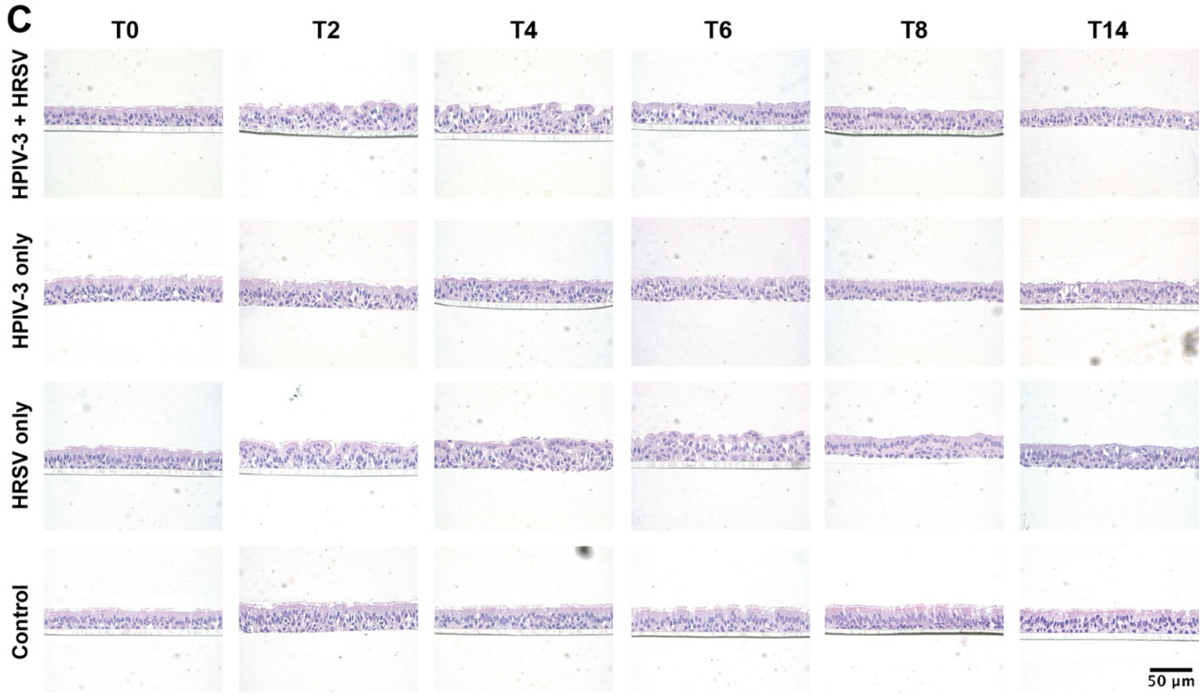

HPIV-3 and/or HRSV infection leads to cilia loss without disrupting epithelial architecture. Cross-sectional images of all transwells over time stained with hematoxylin and eosin (H&E). Abbreviations: HPIV-3: human parainfluenza virus 3, HRSV = human respiratory syncytial virus; T = timepoint.

Supplemental Table 1

Post-hoc Tukey's multiple comparisons of LDH measurements between experimental conditions (HPIV-3 + HRSV, HPIV-3 only, HRSV only, and control) across timepoints.

	**HPIV-3 + HRSV vs. HPIV-3 only**	**HPIV-3 + HRSV vs. HRSV only**	**HPIV-3 + HRSV vs. Control**	**HPIV-3 only vs. HRSV only**	**HPIV-3 only vs. Control**	**HRSV only vs. Control**
**T0**	ns (0,9912)	**** (<0.0001)	**** (<0.0001)	**** (<0.0001)	**** (<0.0001)	ns (0,7051)
**T2**	ns (0,5303)	**** (<0.0001)	**** (<0.0001)	**** (<0.0001)	**** (<0.0001)	* (0,0101)
**T4**	*** (0,0009)	ns (0,2309)	**** (<0.0001)	ns (0,422)	**** (<0.0001)	**** (<0.0001)
**T6**	*** (0,0002)	* (0,0207)	**** (<0.0001)	**** (<0.0001)	**** (<0.0001)	**** (<0.0001)
**T8**	ns (0,8558)	*** (0,0001)	** (0,006)	*** (0,0007)	** (0,0031)	**** (<0.0001)
**T14**	ns (0,9716)	ns (0,9947)	ns (0,0814)	ns (0,9993)	ns (0,1897)	ns (0,2623)

Supplemental Table 2

Post-hoc Tukey's multiple comparisons of qPCR measurements of HRSV genomes between experimental conditions (HPIV-3 + HRSV, HPIV-3 only, HRSV only, and control) across timepoints.

	HPIV-3 + HRSV vs. HPIV-3 only	HPIV-3 + HRSV vs. HRSV only	HPIV-3 + HRSV vs. Control	HPIV-3 only vs. HRSV only	HPIV-3 only vs. Control	HRSV only vs. Control
**T0**	**** (<0.0001)	ns (0,2031)	** (0,0011)	**** (<0.0001)	ns (0,5438)	** (0,0025)
**T1**	**** (<0.0001)	ns (0,7589)	**** (<0.0001)	**** (<0.0001)	()	**** (<0.0001)
**T2**	**** (<0.0001)	**** (<0.0001)	**** (<0.0001)	**** (<0.0001)	ns (0,7519)	**** (<0.0001)
**T3**	**** (<0.0001)	**** (<0.0001)	**** (<0.0001)	**** (<0.0001)	ns (0,7475)	**** (<0.0001)
**T4**	**** (<0.0001)	**** (<0.0001)	**** (<0.0001)	**** (<0.0001)	ns (0,9237)	**** (<0.0001)
**T5**	**** (<0.0001)	* (0,031)	**** (<0.0001)	**** (<0.0001)	()	**** (<0.0001)
**T6**	**** (<0.0001)	ns (0,5491)	**** (<0.0001)	**** (<0.0001)	ns (0,7538)	**** (<0.0001)
**T7**	**** (<0.0001)	** (0,0051)	**** (<0.0001)	**** (<0.0001)	ns (0,9994)	**** (<0.0001)
**T8**	**** (<0.0001)	** (0,0054)	**** (<0.0001)	**** (<0.0001)	ns (0,4744)	**** (<0.0001)
**T11**	ns (0,0551)	ns (0,9181)	ns (0,0551)	ns (0,0505)	()	ns (0,0505)
**T14**	ns (0,1228)	ns (0,7649)	ns (0,1228)	* (0,0217)	()	* (0,0217)

Supplemental Table 3

Post-hoc Tukey's multiple comparisons of qPCR measurements of HPIV-3 genomes between experimental conditions (HPIV-3 + HRSV, HPIV-3 only, HRSV only, and control) across timepoints.

	**HPIV-3 + HRSV vs. HPIV-3 only**	**HPIV-3 + HRSV vs. HRSV only**	**HPIV-3 + HRSV vs. Control**	**HPIV-3 only vs. HRSV only**	**HPIV-3 only vs. Control**	**HRSV only vs. Control**
**T-3**	* (0,0226)	* (0,0166)	ns (0,0732)	**** (<0.0001)	**** (<0.0001)	ns (0,9575)
**T-2**	ns (0,9837)	**** (<0.0001)	**** (<0.0001)	**** (<0.0001)	**** (<0.0001)	()
**T-1**	ns (0,9898)	**** (<0.0001)	**** (<0.0001)	**** (<0.0001)	**** (<0.0001)	()
**T0**	ns (0,9775)	**** (<0.0001)	**** (<0.0001)	**** (<0.0001)	**** (<0.0001)	ns (0,7514)
**T1**	ns (0,8492)	**** (<0.0001)	**** (<0.0001)	**** (<0.0001)	**** (<0.0001)	ns (0,2092)
**T2**	ns (0,9987)	**** (<0.0001)	**** (<0.0001)	**** (<0.0001)	**** (<0.0001)	ns (0,9652)
**T3**	ns (0,4207)	**** (<0.0001)	**** (<0.0001)	**** (<0.0001)	**** (<0.0001)	ns (0,3008)
**T4**	* (0,0272)	**** (<0.0001)	**** (<0.0001)	**** (<0.0001)	**** (<0.0001)	ns (0,6638)
**T5**	ns (0,0542)	**** (<0.0001)	**** (<0.0001)	**** (<0.0001)	**** (<0.0001)	ns (0,9995)
**T6**	** (0,0018)	**** (<0.0001)	**** (<0.0001)	**** (<0.0001)	**** (<0.0001)	ns (0,7538)
**T7**	** (0,0016)	**** (<0.0001)	**** (<0.0001)	**** (<0.0001)	**** (<0.0001)	ns (0,7567)
**T8**	** (0,002)	**** (<0.0001)	** (0,0011)	**** (<0.0001)	*** (0,0005)	ns (0,6355)
**T11**	ns (0,5115)	ns (0,0986)	ns (0,0986)	* (0,0211)	* (0,0211)	()
**T14**	ns (0,7069)	ns (0,2027)	ns (0,2027)	ns (0,0551)	ns (0,0551)	()

Supplemental Table 4

Post-hoc Tukey's multiple comparisons of viral titer measurements of HRSV between experimental conditions (HPIV-3 + HRSV, HPIV-3 only, HRSV only, and control) across timepoints.

	HPIV-3 + HRSV vs. HPIV-3 only	HPIV-3 + HRSV vs. HRSV only	HPIV-3 + HRSV vs. Control	HPIV-3 only vs. HRSV only	HPIV-3 only vs. Control	HRSV only vs. Control
**T1**	* (0,0158)	ns (0,9713)	* (0,0158)	* (0,0292)	()	* (0,0292)
**T2**	** (0,0032)	ns (0,513)	** (0,0032)	* (0,0129)	()	* (0,0129)
**T3**	*** (0,0009)	ns (0,3787)	*** (0,0009)	**** (<0.0001)	()	**** (<0.0001)
**T4**	* (0,014)	* (0,0415)	* (0,014)	*** (0,0005)	()	*** (0,0005)
**T5**	** (0,0075)	ns (0,2517)	** (0,0075)	**** (<0.0001)	()	**** (<0.0001)
**T6**	** (0,0021)	ns (0,6081)	** (0,0021)	*** (0,0004)	()	*** (0,0004)
**T7**	** (0,0022)	ns (0,7801)	** (0,0022)	*** (0,0002)	()	*** (0,0002)
**T8**	** (0,0024)	ns (0,9461)	** (0,0024)	*** (0,0005)	()	*** (0,0005)
**T11**	* (0,0137)	ns (0,998)	* (0,0137)	ns (0,0657)	()	ns (0,0657)
**T14**	ns (0,2755)	ns (0,9988)	ns (0,2755)	ns (0,3545)	()	ns (0,3545)

Supplemental Table 5
Post-hoc Tukey's multiple comparisons of viral titer measurements of HPIV-3 between experimental conditions (HPIV-3 + HRSV, HPIV-3 only, HRSV only, and control) across timepoints.

	**HPIV-3 + HRSV vs. HPIV-3 only**	**HPIV-3 + HRSV vs. HRSV only**	**HPIV-3 + HRSV vs. Control**	**HPIV-3 only vs. HRSV only**	**HPIV-3 only vs. Control**	**HRSV only vs. Control**
**T-2**	ns (0,9994)	ns (0,0619)	ns (0,0619)	ns (0,0688)	ns (0,0688)	()
**T-1**	ns (0,9152)	**** (<0.0001)	**** (<0.0001)	*** (0,0003)	*** (0,0003)	()
**T0**	ns (0,9999)	**** (<0.0001)	**** (<0.0001)	**** (<0.0001)	**** (<0.0001)	()
**T1**	ns (0,9682)	*** (0,0003)	*** (0,0003)	**** (<0.0001)	**** (<0.0001)	()
**T2**	ns (0,5464)	**** (<0.0001)	**** (<0.0001)	**** (<0.0001)	**** (<0.0001)	()
**T3**	ns (0,0553)	**** (<0.0001)	**** (<0.0001)	**** (<0.0001)	**** (<0.0001)	()
**T4**	ns (0,5456)	*** (0,0002)	*** (0,0002)	**** (<0.0001)	**** (<0.0001)	()
**T5**	** (0,0087)	**** (<0.0001)	**** (<0.0001)	**** (<0.0001)	**** (<0.0001)	()
**T6**	** (0,006)	**** (<0.0001)	**** (<0.0001)	**** (<0.0001)	**** (<0.0001)	()
**T7**	* (0,0158)	**** (<0.0001)	**** (<0.0001)	*** (0,0001)	*** (0,0001)	()
**T8**	** (0,0027)	**** (<0.0001)	**** (<0.0001)	**** (<0.0001)	**** (<0.0001)	()
**T11**	ns (0,3134)	* (0,0149)	* (0,0149)	* (0,0489)	* (0,0489)	()
**T14**	ns (0,639)	* (0,0185)	* (0,0185)	** (0,0084)	** (0,0084)	()

Supplemental Table 6

Post-hoc Tukey's multiple comparisons of IFN-β expression levels between experimental conditions (HPIV-3 + HRSV, HPIV-3 only, HRSV only, and control) across timepoints.

	**HPIV-3 + HRSV vs. HPIV-3 only**	**HPIV-3 + HRSV vs. HRSV only**	**HPIV-3 + HRSV vs. Control**	**HPIV-3 only vs. HRSV only**	**HPIV-3 only vs. Control**	**HRSV only vs. Control**
**T-3**	ns (0,9718)	ns (0,9718)	ns (0,9718)	ns (>0.9999)	ns (>0.9999)	ns (>0.9999)
**T-2**	ns (0,9721)	ns (0,9691)	ns (0,9898)	ns (>0.9999)	ns (0,9989)	ns (0,9984)
**T-1**	ns (0,99)	ns (0,9923)	ns (0,993)	ns (0,8853)	ns (0,9052)	ns (>0.9999)
**T0**	ns (0,8479)	ns (0,2886)	ns (0,3145)	ns (0,1278)	ns (0,1385)	ns (0,9995)
**T1**	ns (0,9983)	ns (0,104)	ns (0,1125)	* (0,0337)	* (0,0372)	ns (0,999)
**T2**	ns (0,9905)	ns (0,1594)	ns (0,1614)	ns (0,2476)	ns (0,2497)	ns (>0.9999)
**T3**	ns (0,0602)	ns (0,076)	ns (0,6114)	ns (0,4517)	* (0,0222)	ns (0,0521)
**T4**	ns (0,7422)	* (0,0376)	ns (0,3919)	ns (0,1461)	ns (0,1781)	* (0,0128)
**T5**	ns (0,9982)	ns (0,4556)	ns (0,8276)	ns (0,3759)	ns (0,8119)	ns (0,2358)
**T6**	ns (0,9386)	ns (0,9152)	ns (0,6702)	ns (0,455)	ns (0,9217)	ns (0,0845)
**T7**	* (0,0287)	ns (0,48)	ns (0,9998)	ns (0,8324)	ns (0,1675)	ns (0,6529)
**T8**	ns (0,8002)	ns (>0.9999)	ns (0,9682)	ns (0,6086)	ns (0,3418)	ns (0,921)
**T11**	ns (0,9242)	ns (0,9417)	ns (0,751)	ns (>0.9999)	ns (0,8709)	ns (0,8987)
**T14**	ns (0,9976)	ns (0,9518)	ns (0,6003)	ns (0,9805)	ns (0,6283)	ns (0,841)

Supplemental Table 7

Post-hoc Tukey's multiple comparisons of IFN-λ1 expression levels between experimental conditions (HPIV-3 + HRSV, HPIV-3 only, HRSV only, and control) across timepoints.

	HPIV-3 + HRSV vs. HPIV-3 only	HPIV-3 + HRSV vs. HRSV only	HPIV-3 + HRSV vs. Control	HPIV-3 only vs. HRSV only	HPIV-3 only vs. Control	HRSV only vs. Control
**T-3**	ns (>0.9999)	ns (>0.9999)	ns (>0.9999)	ns (>0.9999)	ns (>0.9999)	ns (>0.9999)
**T-2**	ns (0,9991)	ns (0,9991)	ns (0,949)	ns (>0.9999)	ns (0,9211)	ns (0,9211)
**T-1**	ns (0,9876)	ns (0,7531)	ns (0,7831)	ns (0,461)	ns (0,4925)	ns (0,9464)
**T0**	ns (0,9432)	ns (0,4785)	ns (0,4835)	ns (0,2096)	ns (0,2121)	ns (0,9994)
**T1**	ns (0,9989)	ns (0,0962)	ns (0,0965)	ns (0,086)	ns (0,0863)	ns (>0.9999)
**T2**	ns (0,9998)	ns (0,3355)	ns (0,1198)	ns (0,2683)	ns (0,0907)	ns (0,5027)
**T3**	ns (>0.9999)	ns (0,2925)	ns (0,1079)	ns (0,2986)	ns (0,0925)	*** (0,0001)
**T4**	ns (0,9712)	** (0,0049)	ns (0,061)	ns (0,0513)	ns (0,1856)	** (0,0014)
**T5**	ns (0,7982)	* (0,0291)	ns (0,099)	ns (0,1522)	ns (0,3486)	* (0,0149)
**T6**	ns (0,801)	ns (0,1703)	ns (0,4451)	ns (0,4184)	ns (0,3747)	ns (0,0966)
**T7**	ns (0,0989)	ns (0,0557)	ns (0,209)	ns (0,8443)	* (0,0348)	* (0,0265)
**T8**	ns (0,3644)	ns (0,4476)	ns (0,6855)	ns (0,9789)	ns (0,0722)	* (0,0473)
**T11**	ns (0,8318)	ns (0,9935)	ns (0,7936)	ns (0,6737)	ns (0,4182)	ns (0,8203)
**T14**	ns (0,9723)	ns (0,8851)	ns (0,6556)	ns (0,3892)	ns (0,1281)	ns (0,8855)

Supplemental Table 8

Post-hoc Tukey's multiple comparisons of IFN-λ2,3 expression levels between experimental conditions (HPIV-3 + HRSV, HPIV-3 only, HRSV only, and control) across timepoints.

	**HPIV-3 + HRSV vs. HPIV-3 only**	**HPIV-3 + HRSV vs. HRSV only**	**HPIV-3 + HRSV vs. Control**	**HPIV-3 only vs. HRSV only**	**HPIV-3 only vs. Control**	**HRSV only vs. Control**
**T-3**	ns (>0.9999)	ns (>0.9999)	ns (0,9864)	ns (>0.9999)	ns (0,9864)	ns (0,9864)
**T-2**	ns (0,9998)	ns (0,9647)	ns (0,9934)	ns (0,9808)	ns (0,9884)	ns (0,9238)
**T-1**	ns (>0.9999)	ns (0,9707)	ns (0,9963)	ns (0,9531)	ns (0,9921)	ns (0,9192)
**T0**	ns (0,5842)	ns (0,2432)	ns (0,2429)	* (0,038)	* (0,0379)	ns (>0.9999)
**T1**	ns (0,8886)	* (0,0268)	* (0,0495)	** (0,0021)	** (0,0047)	ns (0,9497)
**T2**	ns (0,9951)	ns (0,1716)	ns (0,2941)	* (0,0263)	ns (0,0813)	ns (0,9706)
**T3**	ns (0,1708)	ns (0,3499)	ns (0,768)	ns (0,9971)	* (0,0174)	ns (0,0932)
**T4**	ns (0,7192)	* (0,0469)	ns (0,915)	ns (0,1976)	ns (0,3772)	* (0,02)
**T5**	ns (0,9119)	ns (0,3754)	ns (0,9956)	ns (0,6468)	ns (0,8561)	ns (0,3389)
**T6**	ns (0,9952)	ns (>0.9999)	ns (0,8791)	ns (0,9764)	ns (0,8647)	ns (0,5199)
**T7**	ns (0,1041)	ns (0,2939)	ns (0,9993)	ns (0,9967)	ns (0,188)	ns (0,3947)
**T8**	ns (0,9261)	ns (0,9994)	ns (0,88)	ns (0,7388)	ns (0,3538)	ns (0,464)
**T11**	ns (0,9998)	ns (0,9922)	ns (0,9096)	ns (0,9963)	ns (0,9138)	ns (0,9602)
**T14**	ns (0,9998)	ns (0,999)	ns (0,9808)	ns (0,9999)	ns (0,98)	ns (0,9893)
